# Supplementary material for: A simulated single ventilator/dual patient ventilation strategy for acute respiratory distress syndrome during the COVID-19 pandemic
Source: R Soc Open Sci. 2020 Aug 24;7(8):200585. doi: 10.1098/rsos.200585 (PMC7481711; doi:10.1098/rsos.200585)
Supplement: See attached description of supplementary materials file [file rsos200585supp14.pdf]

Electronic Supplementary Materials

Data for Plots 3-6(b)

General description

Each CSV file contains the necessary vector data to produce the figure described in its title. Each filename consists of the following structure:

Fig<NUMBER>_<SPLITTER_TYPE>_<FIGURE_DESCRIPTION>_<CONTENTS_INFORMATION>.csv, where:

- NUMBER: figure number (3b, 4b, 5, or 6b)
- SPLITTER_TYPE: standardSplitter or modifiedSplitter
- FIGURE_DESCRIPTION: Description relating to figure caption in manuscript.
- CONTENTS_INFORMTATION: Description of the specific contents of the file.

Thirteen files have been submitted.

Descriptions per files

[Figure 3b (4 files)]

Each file contains the Pressure, Tidal Volume and Flow variables for a specific patient.

- Fig3B_standardSplitter_PressureVolumeFlow_PatientModelsAtoD_LungModelA: Variables for a patient with a lung model A (healthy, compliance at 0.054 L/cmH2O)

- Fig3B_standardSplitter_PressureVolumeFlow_PatientModelsAtoD_LungModelB: Variables for a patient with a lung model B (mild ARDS, 20% reduction with respect to A)

- Fig3B_standardSplitter_PressureVolumeFlow_PatientModelsAtoD_LungModelC: Variables for a patient with a lung model C (moderate, 30% reduction)

- Fig3B_standardSplitter_PressureVolumeFlow_PatientModelsAtoD_LungModelD: Variables for a patient with a lung model D (severe, 40% reduction)

[Figure 4b (3 files)]

Each file contains Pressure, Tidal volume and Flow for two patients paired under the standard splitter.

Fig4B_standardSplitter_comparison_PressureVolumeFlow_dissimilarPatientModels_Patients_A_B: Variables for patient pair A-B.

Fig4B_standardSplitter_comparison_PressureVolumeFlow_dissimilarPatientModels_Patients_A_D: Variables for patient pair A-C.

Fig4B_standardSplitter_comparison_PressureVolumeFlow_dissimilarPatientModels_Patients_A_D: Variables for patient pair A-D.

[Figure 5b (3 files)]

Each file contains Pressure, Tidal volume and Flow for two patients paired under the modified splitter.

`Fig5B_modifiedSplitter_normaliseTidalVolume_dissimilarPatientModels_Patients_A_B`: Variables for patient pair A-B where tidal volume was normalised using the proposed Modified Splitter.

`Fig5B_modifiedSplitter_normaliseTidalVolume_dissimilarPatientModels_Patients_A_C`: Variables for patient pair A-C where tidal volume was normalised using the proposed Modified Splitter.

`Fig5B_modifiedSplitter_normaliseTidalVolume_dissimilarPatientModels_Patients_A_D`: Variables for patient pair A-D where tidal volume was normalised using the proposed Modified Splitter.

[Figure 6b (3files)]

Each file contains Pressure, Tidal volume and Flow for two patients with a lung model C (C-C) paired, where the tidal volume was altered by the modified splitter.

`Fig6B_modifiedSplitter_adjustTidalVolume_Patients_C_C_baseline`: Variables for pair C-C, where no alteration was made.

`Fig6B_modifiedSplitter_adjustTidalVolume_Patients_C_C_decrease`: Variables for pair C-C, where the Tidal Volume of one patient was decreased by 30% whilst maintaining the other patient constant.

`Fig6B_modifiedSplitter_adjustTidalVolume_Patients_C_C_increase`: Variables for pair C-C, where the Tidal Volume of one patient was increased by 30% whilst maintaining the other patient constant.

Data description per column

Each file contains a time column and data columns, each of them with a descriptive heading. Headings contain the following structure:

<VARIABLE>_<PATIENT_PAIR>_<PATIENT>, where:

`VARIABLE`: Control, Pressure (in cmH2O), ModifiedVol (Tidal Volume in mL), and Flow (in L/min)

`PATIENT_PAIR`: Includes all pairwise combinations of letters A,B,C and D (corresponding to the Lung Compliance Models described in the manuscript).

`PATIENT`: Variable corresponding to the specific patient in a pair. For example, `Pressure_AD_1A` corresponds to patient 1, with a lung model A.
